# Particle segmentation algorithm for flexible single particle reconstruction

**DOI:** 10.1007/s41048-017-0038-7

**Published:** 2017-05-19

**Authors:** Qiang Zhou, Niyun Zhou, Hong-Wei Wang

**Affiliations:** 1 State Key Laboratory of Biomembrane and Membrane Biotechnology, Center for Structural Biology, School of Life Sciences, Tsinghua University, Beijing, 100084 China; 2 Ministry of Education Key Laboratory of Protein Science, Tsinghua-Peking Joint Center for Life Sciences, Center for Structural Biology, School of Life Sciences, Tsinghua University, Beijing, 100084 China

**Keywords:** Single particle reconstruction, Cryo-EM, Particle segmentation, Local reconstruction

## Abstract

As single particle cryo-electron microscopy has evolved to a new era of atomic resolution, sample heterogeneity still imposes a major limit to the resolution of many macromolecular complexes, especially those with continuous conformational flexibility. Here, we describe a particle segmentation algorithm towards solving structures of molecules composed of several parts that are relatively flexible with each other. In this algorithm, the different parts of a target molecule are segmented from raw images according to their alignment information obtained from a preliminary 3D reconstruction and are subjected to single particle processing in an iterative manner. This algorithm was tested on both simulated and experimental data and showed improvement of 3D reconstruction resolution of each segmented part of the molecule than that of the entire molecule.

**Electronic supplementary material:**

The online version of this article (doi:10.1007/s41048-017-0038-7) contains supplementary material, which is available to authorized users.

## Introduction

Single particle cryo-electron microscopy (cryo-EM) is a powerful structural biology tool being developed in the past several decades and becoming more matured in recent years (Bai *et al*. 2015a; Carazo *et al*. 2015; Cheng 2015; Cheng *et al*. 2015; Nogales and Scheres 2015). By quickly freezing biological macromolecules in a thin film of vitreous ice, cryo-EM preserves the molecules as they are in solution immediately before the freezing. This stipulates cryo-EM the unique advantage to reveal the molecular structure in their close-to-native states and the possibility to examine structures in action. The most recent development of new direct-electron detection device and image processing algorithms has dramatically boosted the capability of this technique so that three-dimensional (3D) structures of biological macromolecules can be solved to near atomic resolution from averaging many individual images without crystallization (Bai *et al*. 2013; Liao *et al*. 2013; Bartesaghi *et al*. 2015). This has led to a resolution revolution of the cryo-EM technology and is transforming the field of structural biology (Kuhlbrandt 2014).

Despite the major technical progresses, compositional and conformational heterogeneity still imposes a major obstacle on high-resolution single particle cryo-EM structural determination. Different from crystallography where the macromolecules are constrained within a crystalline lattice, single particle molecules in solution are more flexible in changing their ternary and quaternary structures which may cause conformational or compositional heterogeneity among the molecules. In cases where the heterogeneity is relatively subtle and localized, single particle 3D reconstruction of a macromolecule complex is an averaged structure of the common region of all the molecules but with a low resolution at the flexible region. Algorithms based on multivariate statistical analysis were developed to classify molecules into different states (van Heel and Frank 1981). The maximum likelihood algorithm was developed to classify molecule images with low signal to noise ratio (Scheres *et al*. 2007). Methods such as random conical tilt and orthogonal tilt reconstruction were developed to obtain 3D models of different molecular states (Radermacher *et al*. 1987; Leschziner and Nogales 2006). Using statistical classification approach, these algorithms sort the heterogeneous particle images into different classes based on the level of similarity among them and treat each class of images as a homogeneous set of molecules. The classification thus generates multiple structures each reflecting a different state of the biological sample in vitreous ice. The above methods all assume common structure within the same class of molecules. While these methods have been proved to be very successful on the structural studies of many macromolecular complexes and revealed important mechanistic insight to the conformational switch of important molecular machines, there are still a lot of complexes with more complicated conformational heterogeneity that cannot be easily studied. In a severe conformational heterogeneity such as a global variation within the molecule or a continuous domain–domain movement at large scale, a correct 3D reconstruction cannot even be obtained using the conventional classification approach.

Several algorithms without classification strategy have been introduced to single particle analysis of macromolecular complexes with continuous conformational changes. These include the normal-mode analysis (Ma and Karplus 1997; Brink *et al*. 2004; Ma 2005; Jin *et al*. 2014), energy landscape analysis and manifold embedding (Dashti *et al*. 2014; Frank and Ourmazd 2016), 3D variance analysis (Penczek *et al*. 2006; Zhang *et al*. 2008), covariance analysis (Anden *et al*. 2015; Katsevich *et al*. 2015; Liao *et al*. 2015), and eigen analysis-based methods (Penczek *et al*. 2011; Tagare *et al*. 2015). These algorithms can provide quantitative description of the conformational variation mode in the complex to guide further processing of the dataset. More recently, local masking technique was used in reconstructing the rigid body within a complex or further classifying local subtle conformational heterogeneity in a focused region of the molecule. This has been quite successful in improving the local resolution significantly of different rigid portions within a complex (Amunts *et al*. 2014; Brown *et al*. 2014; Chang *et al*. 2015; Yan *et al*. 2015).

Further implementation of algorithms that can separate the relative mobile parts within a flexible molecule and reconstruct the different parts separately will be more useful. Because the electron micrograph of a molecule reflects the 2D projection of the molecule along the electron beam illumination direction, different parts of the complex superimpose with each other in the 2D image. So simply masking the 2D image or 3D model does not eliminate the influence by the signal of the mobile portion on the 3D reconstruction. A clearer way should be to remove the signal of mobile portion from the 2D image entirely so a reconstruction of the interesting part can be done with greater fidelity. Such kind of separation has been realized in Fourier–Bessel space for the reconstruction of a double-layered helical assembly of tubulin (Wang and Nogales 2005). Recently, separation and reconstruction of icosahedral viral genomic structure from the capsid structure were achieved by subtracting the capsid signal from the raw images of virus particles (Liu and Cheng 2015; Zhang *et al*. 2015). In our most recent work, we have developed a segmentation algorithm to separate the SNAP–SNARE structure from 20S particle by subtracting the hexameric NSF complex in the raw image of 20S particle and thus overcome the symmetry mismatch and severe conformational heterogeneity in the 20S particles. This allowed us to reconstruct the SNAP–SNARE complex with higher resolution than using the whole particle images (Zhou *et al*. 2015). At nearly the same time, Bai *et al*. (2015b), Ilca *et al*. (2015), and Shan *et al*. (2016) developed similar algorithms independently. A recent development in RELION software (Scheres 2012a, b) makes it possible to subtract certain portions within a complex from the raw 2D images without introducing major artifact. This allowed much better classification of the interested portion to further sort the heterogeneous particle images to even higher resolution than the overall average (Bai *et al*. 2015b).

In this work, we further expand the particle segmentation algorithm that we have developed for the analysis of 20S particles to other samples. The successful application of this algorithm to different systems with conformational heterogeneity indicated its generality. We also incorporated the image subtraction algorithm at micrograph level so it not only overcomes the potential artifact from interpolation and contrast transfer function, but more importantly also provides new opportunities to analyze micrographs of crowding particle images.

## Theory and algorithm

### Particles segmentation

In the current algorithm, we consider a scenario where the being-studied macromolecule is composed of two rigid bodies that are relatively mobile with each other. In a cubic volume with *N* × *N* × *N* voxels, the 3D densities of the two rigid bodies are *V*_1_ and *V*_2_, respectively. For a certain conformation of the macromolecule, its 3D density *V* thus can be written as

1 $$V = V_{1} \cdot E_{1} + V_{2} \cdot E_{2} ,$$

where *E*_1_ and *E*_2_ are the Euler matrix of *V*_1_ and *V*_2_, respectively. The Euler matrices are functions of Euler angles and translational vectors

2 $$E_{k} = f\left( {\varPhi_{k} ,\overrightarrow {{r_{k} }} } \right),k = 1,2 .$$

The different combinations of *E*_1_ and *E*_2_ define a heterogeneous conformation among the molecules. Our goal is to determine the high-resolution structure of the two rigid bodies, *V*_1_ and *V*_2_. During the process, we should also be able to reveal all the *E*_1_ and *E*_2_ combinations therefore the conformational distribution within the specimen.

For a particle *i* in a transmission electron microscope, its 2D image as a *N* × *N* array is

3 $$X_{i} = F^{ - 1} \left[ {CTF_{i} \cdot \left( {A^{{E_{1,i} }} \cdot F\left( {V_{1} } \right) + A^{{E_{2,i} }} \cdot F\left( {V_{2} } \right)} \right) + N_{i} } \right] ,$$

where *F* and *F* ^−1^ are Fourier transform and reverse Fourier transform operation, respectively; *CTF*_*i*_ is the contrast transfer function for particle *i*; $A^{{E_{k,i} }}$ is the slicing operation on the 3D Fourier transform according to *E*_*k,i*_, *k* = 1,2; *N*_*i*_ is the noise of the particle *i.*

In this 2D image, the projection of *V*_1_ or *V*_2_ is

4 $$P_{k,i} = F^{ - 1} \left[ {CTF_{i} \cdot A^{{E_{k,i} }} \cdot F\left( {V_{k} } \right)} \right],k = 1,2.$$

**Fig. 1:**
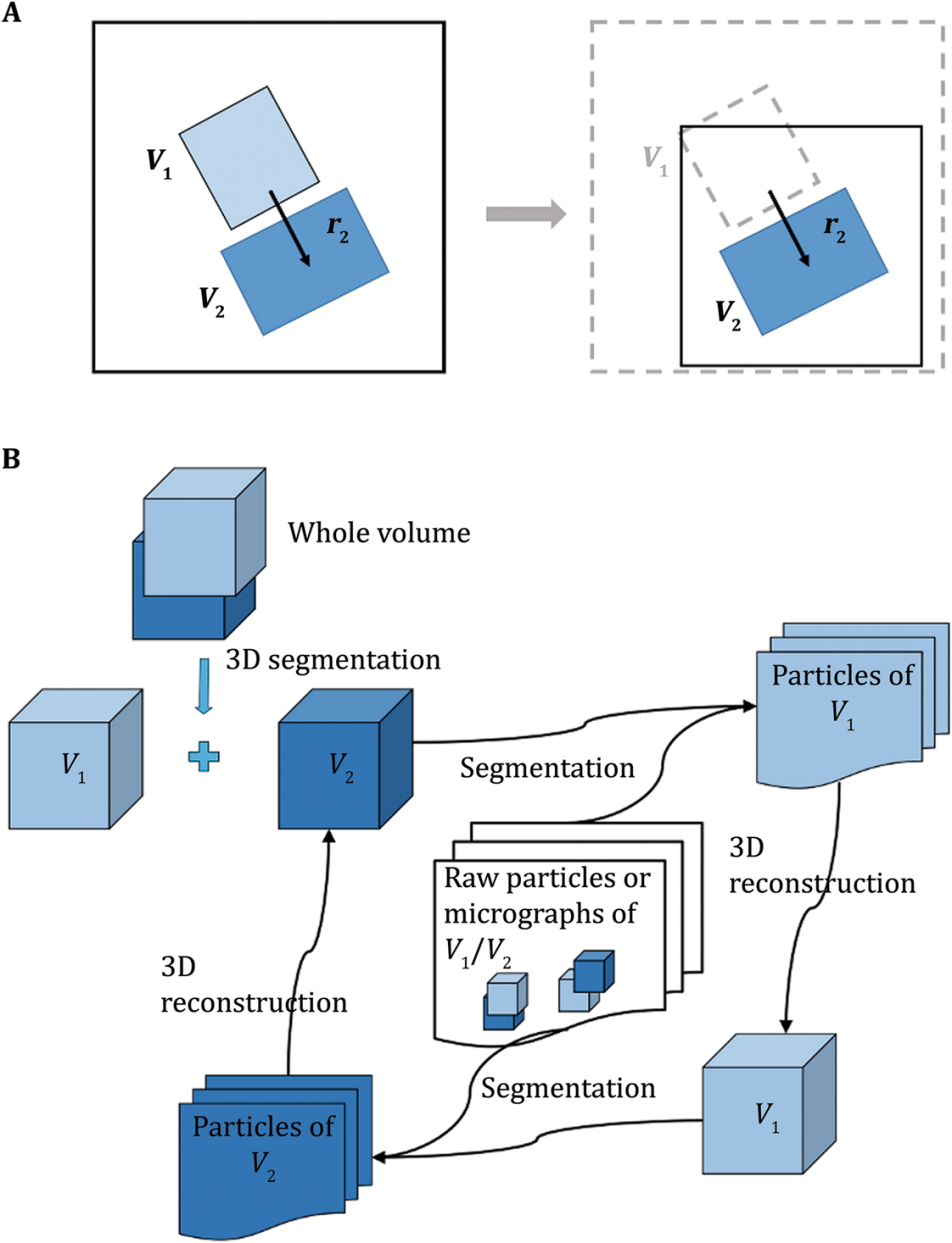
Flowchart of particle segmentation and 3D reconstruction. **A** The *V*_2_ part of a particle is re-windowed and centered from the raw particle image according to its location *r*_2_, meanwhile the *V*_1_ part is subtracted from the raw particle image. **B** The flowchart of iterative segmentation and reconstruction. The raw particles are composed of two rigid parts flexible to each other: *V*_1_ and *V*_2_. Firstly, the whole 3D volume of initial model is segmented into *V*_1_ and *V*_2_. Then *V*_2_ is subtracted from raw particle images or micrographs, from which the *V*_1_ particle images are re-windowed and subjected to 3D reconstruction, resulting in a refined *V*_1_. This process is repeated again with *V*_1_ subtracted from raw particle images or micrographs, obtaining *V*_2_ particle images and a refined *V*_2_. The procedure can be repeated until convergence

If we know *V*_1_ and *V*_2_ and their exact corresponding Euler matrices, we should be able to subtract the signal of either *V*_1_ or *V*_2_ from the raw particle or micrograph and then segment the other part according to its location for further analysis (Fig. 1A).

5 $$X_{h,i} = Win\left( {r_{h,i} ,b} \right)\left( {X_{i} - P_{k,i} } \right),k,h = 1,2,k \ne h ,$$

where $r_{h,i}$ is the location of *V*_*h*_ and $Win\left( {r_{h,i} ,b} \right)$ is a function to re-window an image with box size *b* at $r_{h,i}$, *h* = 1,2. This operation thus calculates a new image with most of the signal of *V*_*k*_ removed.

In situations where the flexibility between the two rigid bodies is within certain range, *i.e*., the 20S particle, a global low-resolution reconstruction from all the images may serve as a starting model. The initial *V*_*k*_ can be obtained from this global reconstruction through 3D segmentation. The initial *E*_*k,i*_ can be roughly estimated as the Euler matrix obtained from the global reconstruction. These initial values can also be obtained by further focused 3D refinement with corresponding local mask applied. The initial location $r_{h,i}$ for *V*_*h*_ can be obtained from its location in the global 3D reconstruction ($r_{3D,h,i}$) and corresponding Euler matrix *E*_*h,i*_

6 $$r_{h,i} = P_{XY} \cdot E_{h,i} \cdot r_{3D,h,i} ,h = 1,2 ,$$

where $P_{XY}$ is an operation to project vector to *XY* plane.

More specifically, we can first subtract *V*_2_ and generate images for *V*_1_. Then we can get an updated volume and Euler matrix for *V*_1_ with which we can generate images for *V*_2_. These procedures can be iterated between *V*_1_ and *V*_2_ for several rounds until convergence (Fig. 1B).

Because the true value of *V*_*k*_ (*V*_*k,true)*_ is unknown and can only be estimated with *V*_*k*_ at the resolution of the 3D reconstruction, the projection subtracting residual should be:

7 $$\Delta P_{k,i} \approx F^{ - 1} \left[ {CTF_{i} \cdot A^{{E_{k,i} }} \cdot \left. {F\left( {V_{k,true} } \right)} \right|_{{R > R_{k} }} } \right],k = 1,2 ,$$

where *R* is spatial frequency and *R*_*k*_ is the 3D reconstruction resolution. If the initial estimated volume function of *V*_*k*_ can be of enough high resolution, the intensity of *∆P*_*k,i*_ can be neglected.

## Results

### Segmentation algorithm improves the resolution of simulated 20S particle dataset

**Fig. 2:**
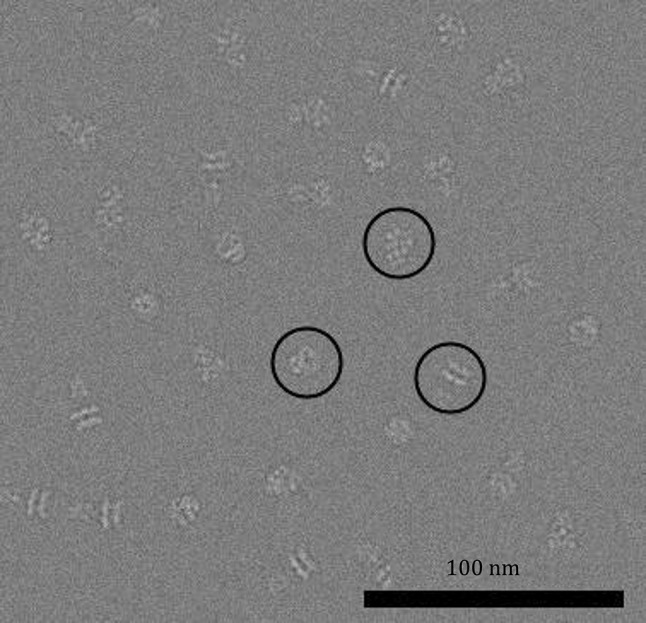
An area of simulated micrograph. Three simulated 20S particles in various views are marked by circles

**Fig. 3:**
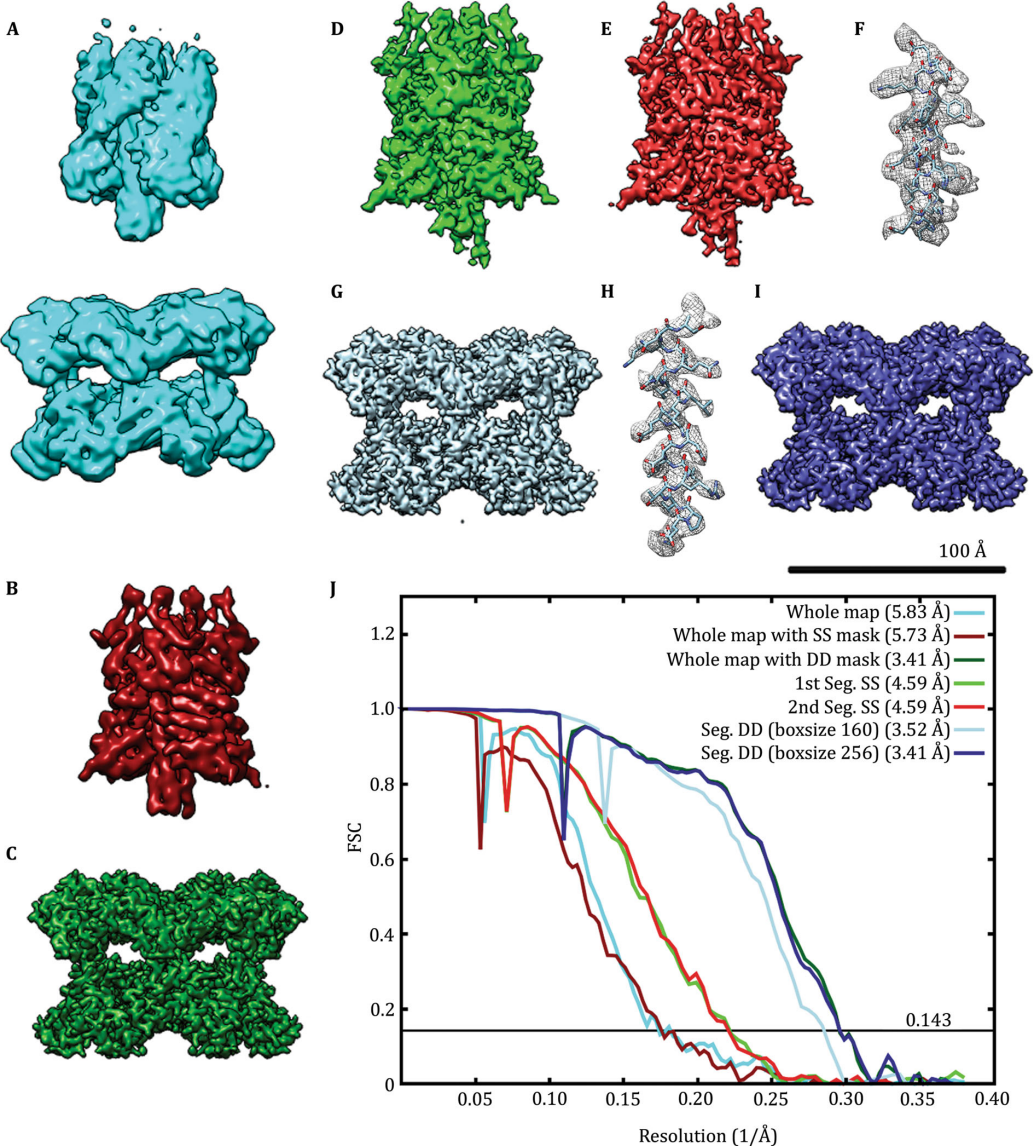
Comparison of 3D reconstructions from simulated 20S particles. **A** 3D reconstruction of whole particles without local mask. **B** 3D reconstruction of whole particles with a local mask around the SS portion. Only SS is shown. **C** 3D reconstruction of whole particles with a local mask around the DD portion. Only DD is shown. **D** 3D reconstruction of the SS particles after the first round of segmentation. **E** 3D reconstruction of the SS particles after the second round of segmentation. **F** An *α*-helix from the 3D density of **E** with the corresponding atomic model docked in. This corresponds to the amino acid residues 138–156 of the *α*-SNAP. **G** 3D reconstruction of the DD particles after the first round of segmentation. **H** An *α*-helix from the 3D density of **G** with the corresponding atomic model docked in. This corresponds to the amino acid residues 511–531 of the NSF. **I** 3D reconstruction of the DD particles after the first round of segmentation with a box size of 256 pixels. **J** FSC curves of the 3D reconstructions. The FSC curve of segmented SS is the one after the second round of segmentation

From the 48 simulated micrographs of 20S particles (Fig. 2, Table 1 for simulating parameters), we extracted the 20S particle images and performed 2D classification and 3D reconstruction of the whole particle images. These showed overall shape of the 20S particle comprising two fuzzy parts corresponding to the SNARE/SNAP (SS) and the D1–D2 domain of NSF (DD), respectively (Supplementary Fig. S1A, Fig. 3A). While the FSC of this overall reconstruction reported a resolution of 5.8 Å, the EM map lacks clear features especially in the SS region. We performed additional 3D reconstruction refinements with local masks around SS or DD, resulting in slightly better-defined SS at 5.7 Å resolution (Fig. 3B) and much better DD at 3.4 Å resolution (Fig. 3C), respectively. The 3D auto-refinements with sub-particles generated with relion_project resulted in similar resolution of 5.45 Å for SS and 3.35 Å for DD (Supplementary Fig. S2, Table 2). Alternatively, we applied the segmentation algorithm to the dataset (Supplementary Fig. S1B–D) and obtained a better-defined reconstruction of SS than the previous two SS volumes at 4.59 Å resolution even in the first round of segmentation (Fig. 3D). After second round of segmentation, the map quality was further improved (Fig. 3E, F) although the apparent FSC value didn’t change significantly from the first round reconstruction (Fig. 3J). The segmentation algorithm also resulted in a DD (Fig. 3G–I, Supplementary Fig. S1E) better than those in the overall 3D reconstruction.

**Table 1:** Parameters for micrograph simulation

Cs (mm)	Voltage (kV)	Defocus range (µm)	Astigmatism (Å)	Amplitude contrast	B factor (Å^2^)	Pixel size (Å/pixel)	*σ* of translation between SS and DD (pixel)	*σ* of Euler angle difference between SS and DD (°)
2.7	300	−1 to −3	1000 ± 50	0.1	50 ± 2	1.32	2	10

**Table 2:** Summary of 3D reconstruction

	Resolution before post-processing	Resolution after post-processing	Symmetry	# Particles	Box size (pixel)
Whole volume of simulated particles	9.13	5.83	C1	7193	256
Whole volume of simulated particles with SS mask	8.24	5.73	C1	7193	256
Whole volume of simulated particles with DD mask	4.12	3.41	C6	7193	256
SS sub-particles generated with relion_project	7.86	5.45	C1	7193	256
DD sub-particles generated with relion_project	4.12	3.35	C6	7193	256
Segmented SS particles round I	6.40	4.59	C1	7163	160
Segmented DD particles (box size 160)	3.91	3.52	C6	7157	160
Segmented DD particles (box size 256)	4.33	3.41	C6	7157	256
Segmented SS particles round II	6.21	4.59	C1	7157	160
Whole volume of 70S ribosome	3.93	3.45	C1	68,543	280
Whole volume of 70S ribosome with 50S mask	3.81	3.16	C1	68,543	280
Whole volume of 70S ribosome with 30S mask	4.20	3.39	C1	68,543	280
50S ribosome generated with relion_project	3.81	3.16	C1	68,543	280
30S ribosome generated with relion_project	4.25	3.36	C1	68,543	280
Segmented 30S subunit	4.20	3.33	C1	68,543	280
Segmented 50S subunit	3.81	3.19	C1	63,499	280
Whole volume of influenza RdRP tetramer	7.68	7.51	C2	67,066	256
Whole volume of influenza RdRP tetramer with dimer mask	6.38	4.33	C2	67,066	256
Segmented influenza RdRP dimer	4.95	4.32	C2	122,758	180
Influenza RdRP dimer generated with relion_project	6.14	4.45	C2	134,132	256

It is notable that the image box size of the windowed particle has an effect on the reconstruction resolution of DD particles. The 3D reconstruction resolution of the segmented DD with a box size of 160 and 256 pixels was 3.52 Å and 3.41 Å, respectively (Fig. 3G, I, J, Table 2). Because the signal of particles is proportional to the molecular weight and the noise is proportional to the box size (Rosenthal and Henderson 2003), using too large box size will decrease the signal to noise ratio of particles. But on the other hand the too small box size results in too large reciprocal pixel size, which may limit the CTF correction and interpolation in Fourier space (Penczek *et al*. 2014). The optimal box size used for 3D reconstruction may be variable for particles with different sizes and/or symmetry.

### Segmentation algorithm improves the reconstruction quality of influenza RdRP

**Fig. 4:**
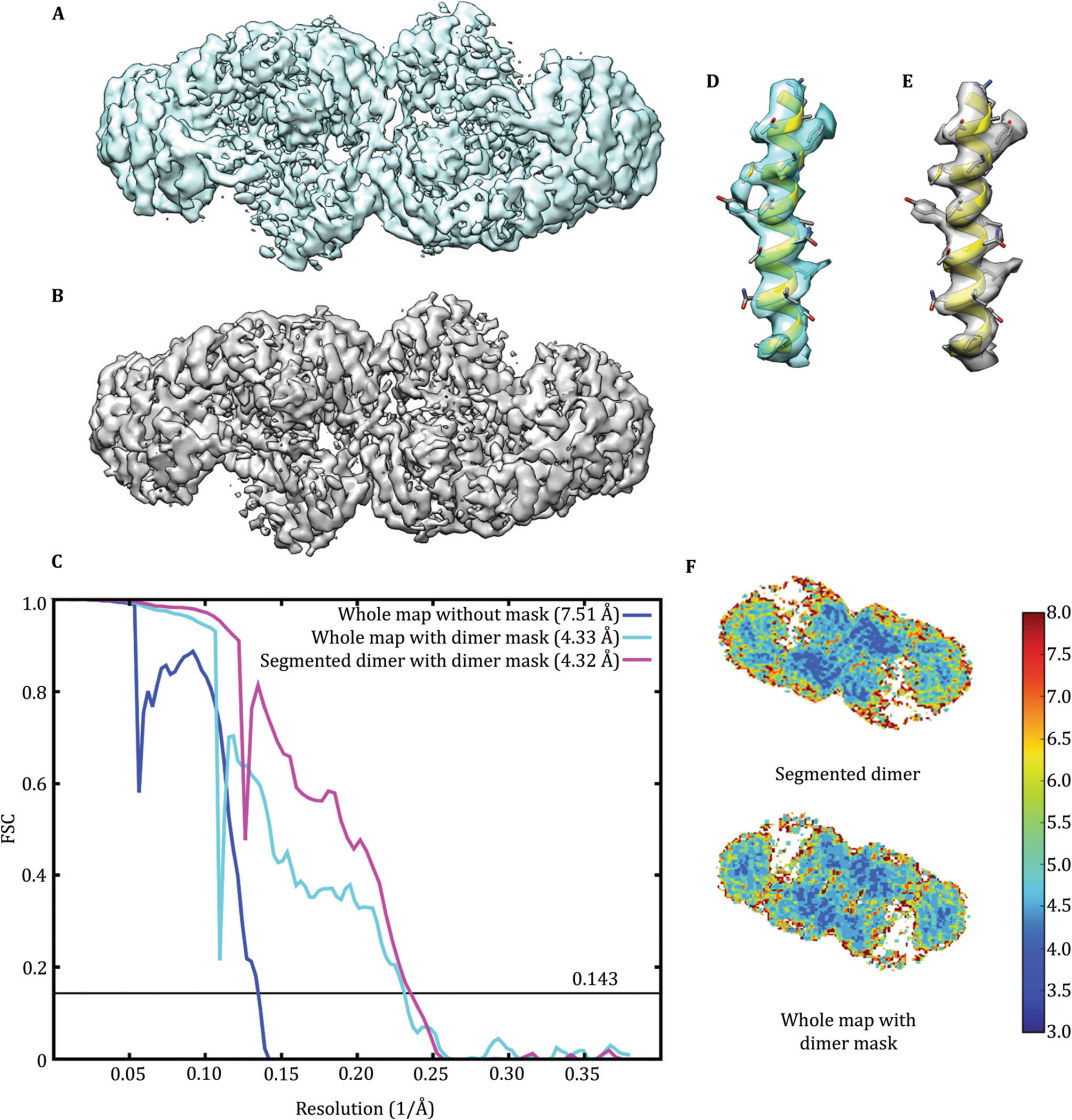
Comparison of 3D reconstructions of influenza RdRP. **A** 3D reconstruction of influenza RdRP tetramer particles with a local mask around the dimer portion (EMD ID: 6202). **B** 3D reconstruction of the influenza RdRP dimer after the first round of segmentation from the tetramer particle images. **C** FSC curves of 3D reconstructions. **D** and **E** Enlarged views of an α-helix density with the corresponding atomic models from **A** and **B**, respectively. The α-helix corresponds to the amino acid residue 454–476 of polymerase basic protein 1 of RdRP. **F** Central slice of the maps colored by local resolution computed with ResMap

Our previous work has shown that the influenza RdRP tetramer contains two homo-dimers interacting with each other in a flexible manner (Chang *et al*. 2015). We were able to obtain a 3D reconstruction of the RdRP dimer at resolution of 4.3 Å by applying a mask around one of the dimer density during the refinement (Fig. 4A). In this practice, each particle image lost half of its structural information in the final reconstruction. The segmentation algorithm provides the opportunity to include the other dimer in the final 3D reconstruction thus double the effective dataset. We segmented the RdRP dimers from all the tetramer dataset and performed 2D classification (Supplementary Fig. S3) and 3D refinement. The 3D reconstruction obtained in this way showed a similar apparent resolution as the previous one (Fig. 4B). But closer look at the FSC curves indicated an elevated signal at medium-resolution range from 10 to 5 Å^−1^ in the latter reconstruction (Fig. 4C). The EM density obtained by the segmentation reconstruction algorithm showed better-defined feature and higher local resolution than that obtained by the local masking reconstruction algorithm (Fig. 4D–F). As a control, the 3D auto-refinements with dimer sub-particles generated with relion_project also resulted in similar resolution of 4.45 Å (Supplementary Fig. S4, Table 2).

### Segmentation algorithm calculates conformational flexible distribution of 70S ribosome

**Fig. 5:**
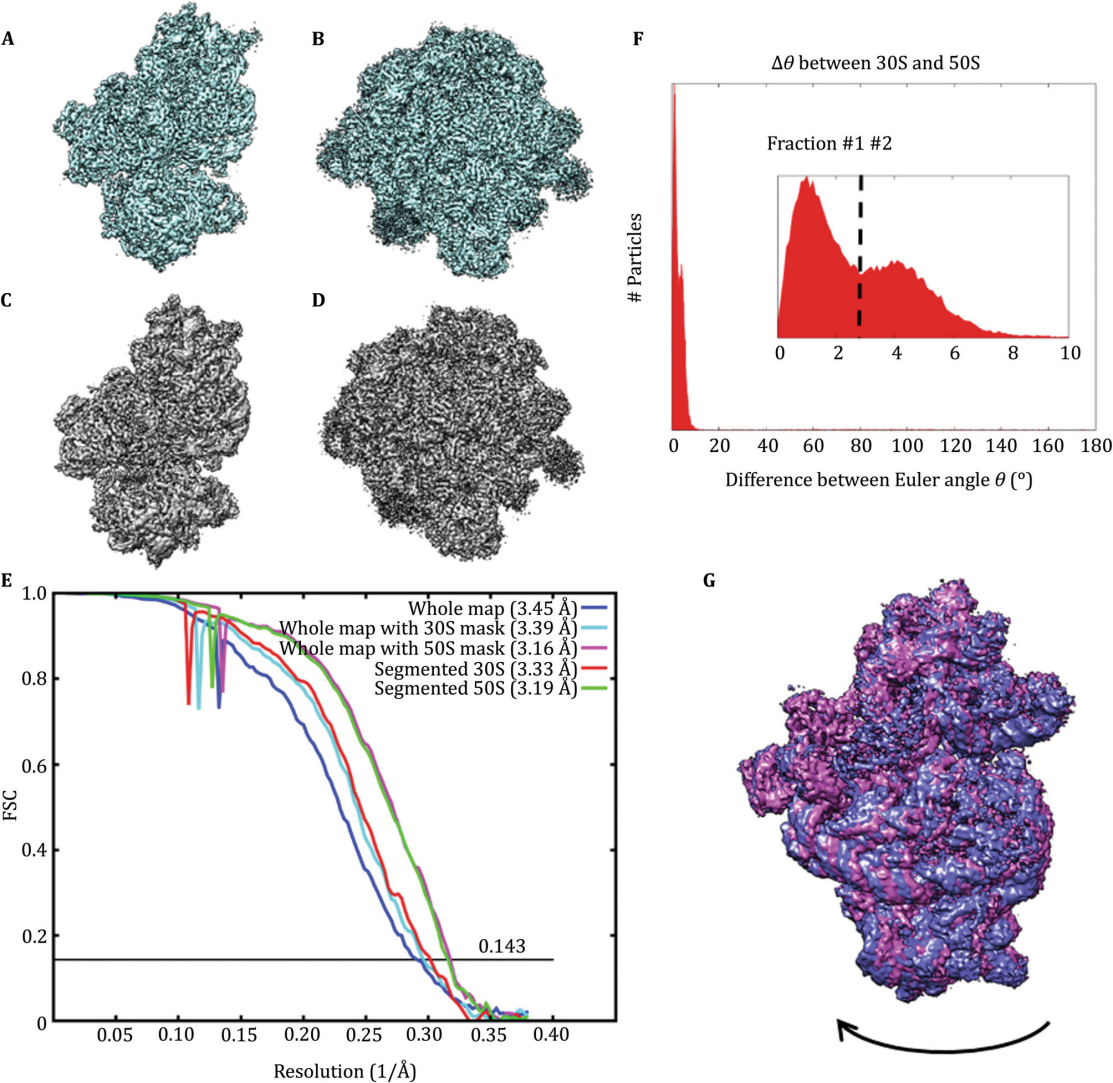
Comparison of 3D reconstructions of 70S ribosome. **A** and **B** are the 3D reconstruction maps of 70S ribosome particles with a local mask of 30S and 50S, respectively. **C** and **D** are the 3D reconstruction maps of 30S and 50S ribosomes after the particle segmentation, respectively. **E** FSC curves of 3D reconstructions. **F** Distribution of the difference of Euler angle theta between the 30S and 50S subunits. Inset is an enlarged view corresponding to the range of theta from 0° to 10°. **G** Comparison between 30S subunit of the 70S ribosome 3D reconstructed from dataset fraction #1 (*blue*) and fraction #2 (*purple*) using the alignment parameters from the 3D auto-refinement of segmented 50S subunit

It is well-known that there is a ratchet motion between the 30S and 50S subunits within a 70S ribosome. Former analysis of 70S ribosomes using supervised classification, maximum likelihood classification, and local masking reconstruction can all separate the different conformers and reconstruct the 30S and 50S portions of the complex. We tested the segmentation algorithm in separating and reconstructing the two portions of 70S ribosome. As a control, we firstly performed 3D reconstruction of the entire 70S particle images and obtained a structure at 3.4 Å resolution. Using local masking approaches, the 30S and 50S subunits can be further refined to 3.4 Å and 3.2 Å resolutions, respectively (Fig. 5A, B). We applied the segmentation algorithm on the dataset and reconstructed the 30S and 50S subunits separately, resulting in final reconstructions at 3.3 Å and 3.2 Å resolutions, respectively (Fig. 5C, D). The 3D auto-refinements with sub-particles generated with relion_project also resulted in similar resolution of 3.4 Å for 30S and 3.2 Å for 50S (Supplementary Fig. S5, Table 2). In summary, both the local masking refinement and segmentation algorithm improved the resolution than the whole particle refinement procedure (Fig. 5E). For both 30S and 50S subunits, the 3D reconstructions using local masking refinement and segmentation algorithm have very similar resolution (Fig. 5E). The reason that there was no improvement is probably due to the rather small motion between the 30S and 50S subunits for which local masking in an auto-refinement obviously restored the orientation of the subunits effectively.

Because we were using segmentation reconstruction, we could calculate the relative rotating angles between 30S and 50S subunits for each individual particle by comparing their Euler angles after the reconstructions. The distribution of the rotation angles showed two peaks, in agreement to the fact that there are two major populations of conformers in the ratchet switch of the 70S ribosome (Fig. 5F). When we aligned the two classes of 3D reconstructions of 70S ribosome based on the 50S subunit, the 30S subunit has a rotation of about 3.8°(Fig. 5G).

### Direct segmentation of particle images from raw micrographs

**Fig. 6:**
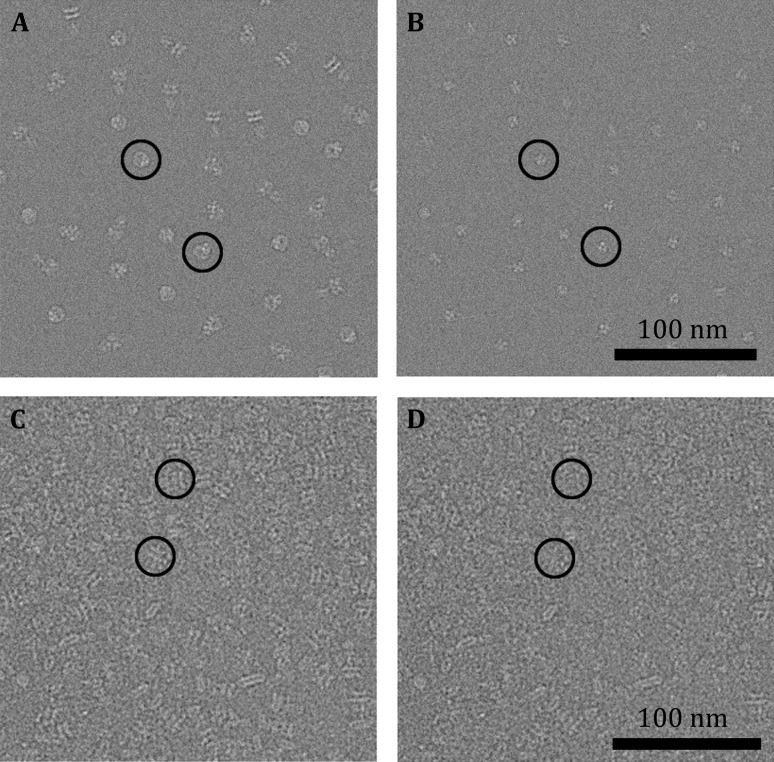
Particle segmentation from raw micrographs. **A** An area of simulated micrograph of the 20S particles. **B** The same micrograph in **A** from which DD particles were subtracted. **C** An area of a raw micrograph of 20S particles. **D** The same micrograph in **C** from which the 20S particles were subtracted. Some typical particles are marked with black circles

We noted that the segmentation algorithm can be directly applied to segment particle images from raw micrographs. As we have discussed previously, the segmentation of raw particle images may suffer from the loss of information due to the point spread function caused by the CTF. After aligning each of the raw particle images with the reference calculated from the partial volume, we should be able to subtract reference projections from the raw micrographs directly. Because there is no cutoff of the CTF fringes around the raw particle images in the whole micrograph, we don’t need to worry about the information loss caused by the windowing. In our simulated micrographs, we can easily subtract the projections of DD from each of the 20S particles (Fig. 6A, B). This can also be done in a real electron micrograph that contains relatively crowded 20S particle images (Fig. 6C, D). This provided opportunities for processing of wider range of cryo-electron micrographs.

## Discussion

Sample heterogeneity is still a major technical obstacle in single particle cryo-EM 3D reconstruction. The source of heterogeneity includes but is not limited to the following aspects: compositional diversity and conformational flexibility. The conformational variation that molecules undergo can be continuous or discrete. Compositional heterogeneity and conformational heterogeneity with discrete states usually lead to a finite number of classes that current 3D classification algorithms can handle reasonably well. In contrast, continuous conformational change within a molecule would lead to an almost infinite number of classes.

3D refinement and reconstruction with an adaptive local mask around the relatively rigid portion of the molecule has shown to be successful in some cases to solve high-resolution structure of certain part of the whole molecule. But in most cases, the overlapped structures in 2D projections interfere correct alignment of the common portion of the molecule. Using the particle segmentation algorithm, we can separate the relatively mobile portions within a molecule image and thus perform single particle analysis of the separated portions without the interference from each other. The image after segmentation has much cleaner signals for more precise alignment and further analysis. Our example of the 20S particle analysis presented in this work indicates the particular advantage of segmentation algorithm in analyzing complexes with internal symmetry mismatch. The further refinement with local angular searching may result in artifact in some cases. In the example of simulated 20S particle, the asymmetric feature of SS part was lost after local angular searching. However, this feature can be well recovered by the segmentation algorithm.

In our segmentation algorithm, after projecting the 3D partial density, it is critical to subtract the projection from raw particles with correct operation. There have been several attempts (Wang and Sigworth 2009; Bai *et al*. 2015b; Ilca *et al*. 2015; Liu and Cheng 2015; Zhang *et al*. 2015) to subtract the projection of a 3D reconstruction or 3D model from raw particles. We found that the absolution gray scale feature of the 3D reconstruction within RELION makes the subtraction easy and intuitive. This operation, which removes most of the low frequency signals of one macromolecule part from the raw particle images, immediately allows the alignment of the other macromolecule part more precisely. This is proved by the fact that reference-free 2D classes of segmented particles show more detailed features than the entire particle but are free of contaminated features from the subtracted references. Furthermore, while we can use the iterative approach (Fig. 1B) to improve the segmentation and alignment of each portion of the molecule, at most two iterations are enough to result the convergence of the solutions in practice (Table 2). This proved that our approximation in Eq. 7 is reasonable for practical purpose.

Besides solving the high-resolution structure of each compositional rigid parts of a complex, the segmentation algorithm provides additional information of the spatial relationship between the rigid parts within each individual particle image. Although in the examples of this work, we mainly focused at the molecules made of two rigid components, the concept can be extended to molecules composed of three or even more rigid bodies that are mobile to each other. Such information of the whole dataset can then be summarized for statistical analysis to reflect the distribution of various conformational states within the flexible molecule. The conformational distribution is of important biological relevance beyond what the static structure can provide, thus realizing the unique power of single particle analysis.

## Materials and methods

### Computation implementation

**Fig. 7:**
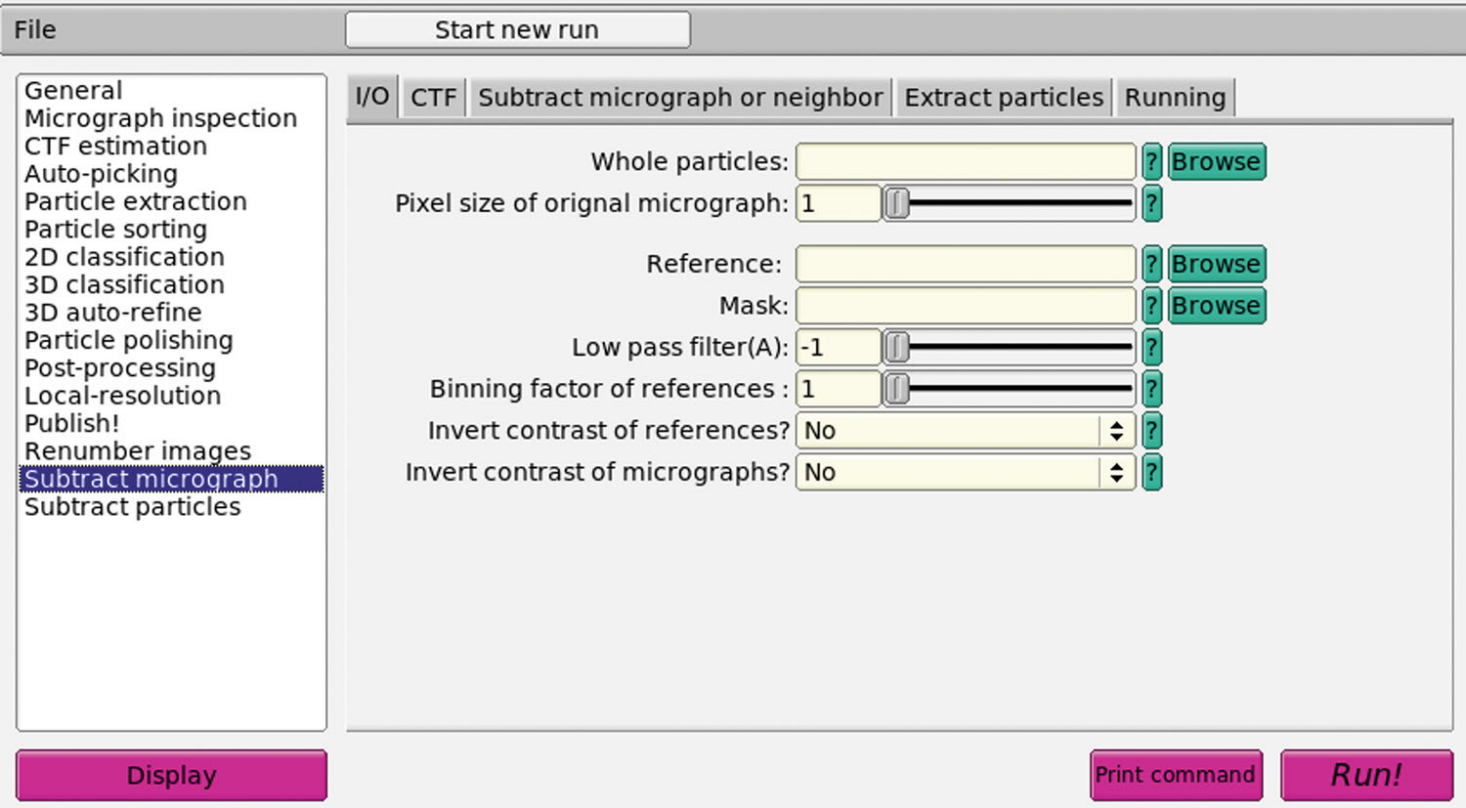
The GUI interface of the segmentation algorithm embedded in RELION package. The segmentation algorithm was embedded in RELION

The particle segmentation algorithm described above was implemented as a new program “subtract_micrograph” and its mpi version “subtract_micrograph_mpi” within the RELION 1.4 package. Part of the source code was copied or adapted from RELION 1.3 or 1.4. We also incorporated this program in a GUI version of RELION 1.4 (Fig. 7).

### Generation of simulated dataset

Previous works (Zhao *et al*. 2015; Zhou *et al*. 2015) showed that human 20S particle functioning in membrane fusion processes in eukaryotic cells is composed of two parts relatively flexible to each other: the SS complex with pseudo four-fold symmetry and the hexameric NSF complex. We used the 20S particle as a testing model to generate simulated dataset. For convenience of the simulation, we built a model of the SS complex without symmetry and a hexameric model of DD imposed with a C6 symmetry using the Modeller software package (Eswar *et al*. 2006). The two atomic models were converted to MRC format with e2pdb2mrc.py in EMAN2 package (Tang *et al*. 2007). The two MRC volumes with voxel size of 1.32 Å representing the SS and DD portions of 20S particle were then assembled together to resemble the overall architecture of 20S particle. Heterogeneous conformational states were generated by randomly tilting the two portions independently with a standard deviation of 10° for all three Euler angles and translating the two parts with a standard deviation of 2 pixels in coordinates. Subsequently, we used the full set of simulated 3D MRC volumes to generate simulated electron micrographs using a program genRandomImage.py written with EMAN2 package. A total of 48 simulated electron micrographs each containing 150 particle images at random orientations and locations were generated. In each of these micrographs, CTF-independent Gaussian white noise was superimposed and CTF-dependent water noise was generated by randomizing the Fourier phase of the atomic model of water molecules simulated with NAMD and VMD (Humphrey *et al*. 1996). The noise level and CTF parameters in these simulated micrographs were chosen to mimic the real micrographs obtained by a Gatan K2-Summit electron counting camera on a Titan Krios microscope operated at 300 kV. More details of the parameters for simulation are listed in Table 1.

### Processing of simulated dataset

A total of 7200 SS/DD particle images were extracted from simulated micrographs with a box size of 256 pixels. These particle images were first 3D refined with RELION 1.3 against an initial model of 20S particle low-pass filtered at 60 Å resolution. As a control, we refined the 3D reconstruction with local angular search range of 30°, during which a SS or DD mask was applied, resulting in a SS or DD volume, respectively. As another control, we also generated SS or DD sub-particles with relion_project and performed 3D auto-refinement with these sub-particles with a local angular search range of 30°. Alternatively, using our implemented segmentation algorithm, the SS particles were segmented by subtracting the DD density from the whole particle images. The segmented and re-windowed SS particles with a box size of 160 pixels were subjected to 2D classification to select the good SS particle images for further 3D refinement in RELION 1.3. After the 3D refinement of segmented SS particles, DD particles were segmented and re-windowed from the whole particle images by subtracting the SS density calculated from the new SS 3D volume. The DD particle images were then subjected to 2D classification and 3D refinement, resulting in an updated DD 3D volume, which was then used for the next cycle of SS segmentation and 3D reconstruction.

### Processing of influenza RdRP

The 3D reconstruction of influenza RdRP tetramer and dimer was described previously (Chang *et al*. 2015). The RdRP dataset from the previous work was used in this study. Each raw particle image containing a tetramer has a pixel size of 1.32 Å and a dimension of 256 pixels. Two RdRP dimer particles were segmented and re-windowed from each raw tetramer particle image with a box size of 180 pixels. Therefore, the particle number of RdRP dimer was doubled after segmentation from the tetramers. The segmented RdRP dimer particles were subsequently used for 2D classification and 3D refinement analysis. As a control, we also generated dimer sub-particles with relion_project and performed 3D auto-refinement with all of the dimer sub-particles.

### Processing of 70S ribosome

We used a cryo-EM dataset of 70S ribosome comprising 68,543 particle images with box size of 280 pixels and a pixel size of 1.32 Å from Prof. Ning Gao’s group. These micrographs were taken from a Titan Krios microscope equipped with a Gatan K2-Summit electron counting camera. We firstly reconstructed a 3D volume of the entire 70S ribosome following the conventional way. This 3D reconstruction was further refined with a local angular search range of 15°, during which a 30S or 50S mask was applied, resulting in the 3D map of 30S or 50S subunit, respectively. We then segmented the 30S subunit from the dataset with a box size of 280 pixels by subtracting the 50S subunit with the segmentation algorithm. The segmented 30S particles were subjected to 2D classification to select good particles for further 3D auto-refinement. The 50S subunit was subsequently segmented from the 70S ribosome images by subtracting the 30S signal using the segmentation algorithm. The segmented 50S subunit images were then refined to reconstruct a 3D volume. As a control, we also generated 30S or 50S sub-particles with relion_project and performed 3D auto-refinement with these sub-particles. The rotating angles between segmented 30S and 50S subunits were calculated with a program CompareDataStars_data.py written with EMAN2 package.

### Other procedures

The micrograph of 20S particle was obtained as described in our previous paper (Zhou *et al*. 2015). 2D classification, 3D reconstruction, and auto-refinement were performed with RELION 1.3. CTF parameters were determined with CTFFIND3 (Mindell and Grigorieff 2003). Reconstruction resolution was estimated with high-frequency noise substituted gold-standard FSC (Scheres and Chen 2012; Chen *et al*. 2013). Local resolution was calculated with ResMap (Kucukelbir *et al*. 2014). Corresponding masks were also applied during the 3D auto-refinement of the segmented particles if not particularly indicated. 3D volume segmentation and atomic model docking were performed with UCSF Chimera (Pettersen *et al*. 2004). The 3D refinements mentioned above are summarized in Table 2.

## Electronic supplementary material

Below is the link to the electronic supplementary material.

Supplementary material 1 (PDF 1071 kb)
