# Supplementary material for: Particle segmentation algorithm for flexible single particle reconstruction
Source: Biophys Rep. 2017 May 19;3(1):43–55. doi: 10.1007/s41048-017-0038-7 (PMC5515998; doi:10.1007/s41048-017-0038-7)
Supplement: Supplementary file 1 — Supplementary material 1 (PDF 1071 kb) [file 41048_2017_38_MOESM1_ESM.pdf]

Supplementary Figures

Particle segmentation algorithm for flexible single particle reconstruction

Qiang Zhou[1,2, §,*], Niyun Zhou[2,3], Hong-Wei Wang[2,*]

[1]State Key Laboratory of Biomembrane and Membrane Biotechnology, Center for Structural Biology, School of Life Sciences, Tsinghua University, Beijing 100084, China

[2]Ministry of Education Key Laboratory of Protein Science, Tsinghua-Peking Joint Center for Life Sciences, Center for Structural Biology, School of Life Sciences, Tsinghua University, Beijing 100084, China

[§]These authors contribute to this work equally.

[**]Correspondence: zhouqiang00@tsinghua.org.cn (Q. Zhou) and hongweiwang@tsinghua.edu.cn (H. Wang)

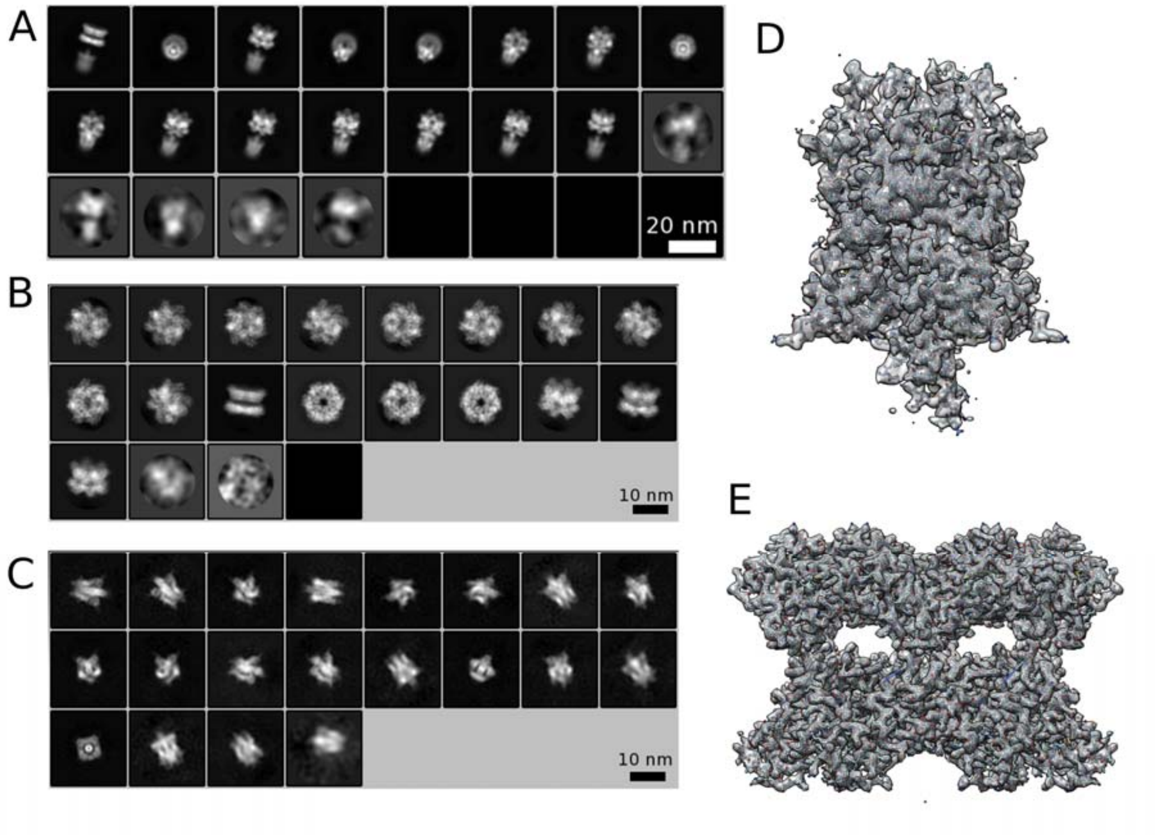

Fig. S1 2D classification, 3D reconstruction and atomic model docking of simulated dataset. **A** 2D classification of the simulated 20S particles. **B** 2D classification of the DD particles after the segmentation to subtract off the SS portion. **C** 2D classification of the SS particles after the first round of segmentation to subtract off the DD portion. **D** Docking of the atomic model of SS into the 3D reconstruction map of the second-round segmented SS particles. **E** Docking of the atomic model of DD into the 3D reconstruction map of segmented DD particles with a box size of 160

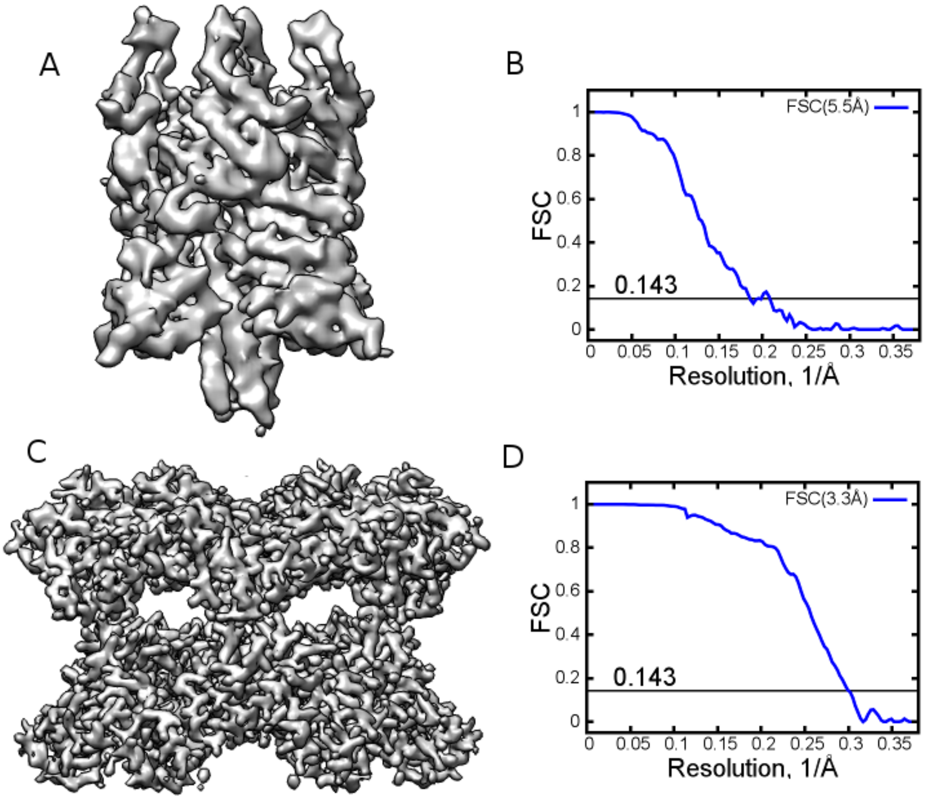

Fig. S2 3D reconstruction of sub-particles generated with relion_project. **A** and **C** are 3D reconstruction maps of SS or DD sub-particles, respectively. **B** and **D** are the FSC curves of the 3D reconstructions of **A** and **C**, respectively

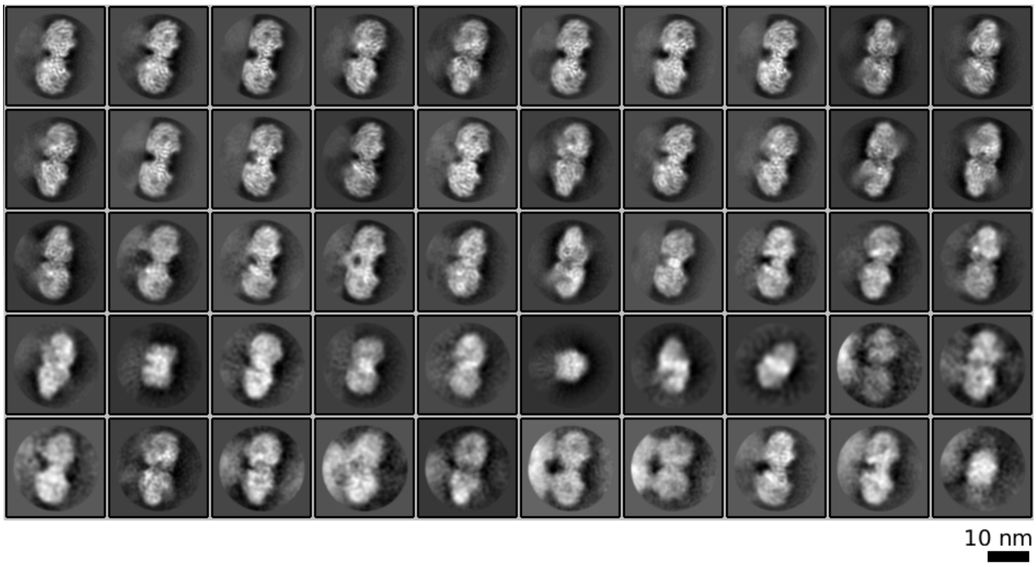

Fig. S3 2D classification of segmented influenza RdRP dimer particles. The 2D class averages are sorted based on the alignment accuracy within each class from the upper-left corner

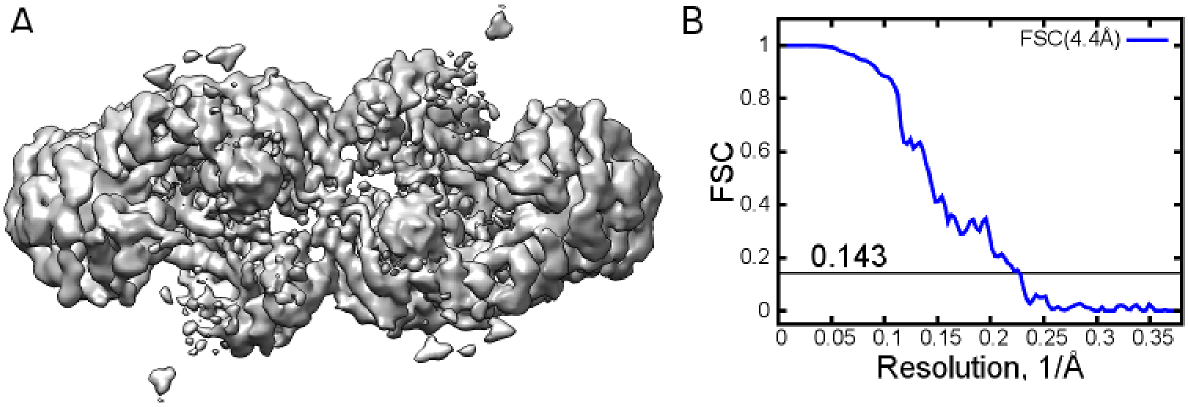

Fig. S4 3D reconstruction of RdRP dimer sub-particles generated with relion_project. **A** is the 3D map and **B** is the FSC curve

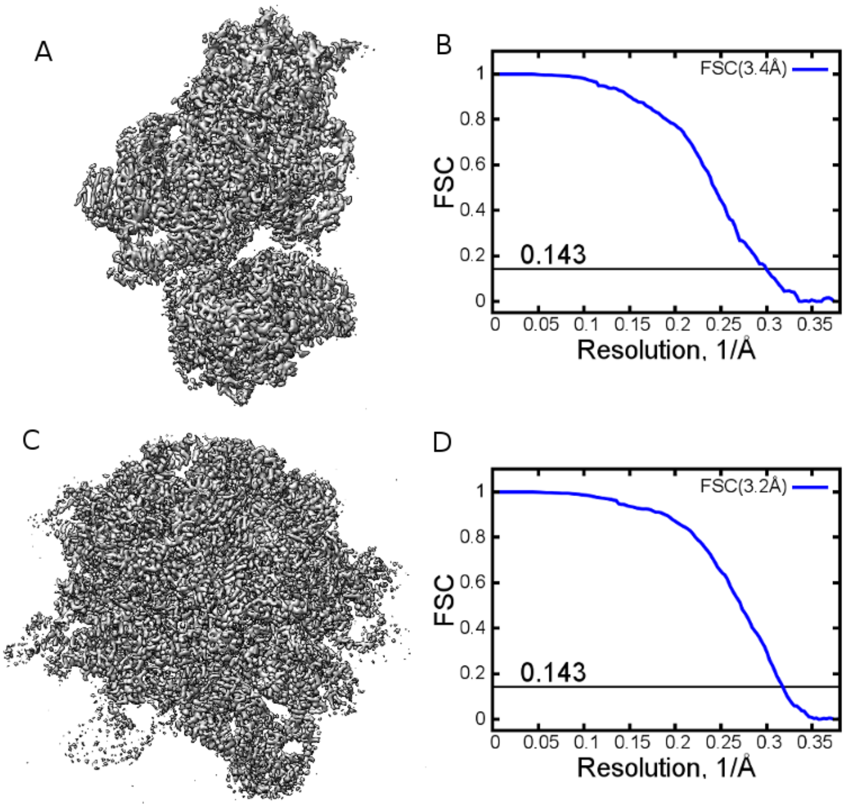

Fig. S5 3D reconstruction of 30S and 50S sub-particles generated with relion_project. **A** and **C** are 3D reconstruction maps of 30S or 50S sub-particles, respectively. **B** and **D** are the FSC curves of the 3D reconstructions of **A** and **C**, respectively
